# Neural Correlates of Workload Transition in Multitasking: An ACT-R Model of Hysteresis Effect

**DOI:** 10.3389/fnhum.2018.00535

**Published:** 2019-01-24

**Authors:** Na Young Kim, Russell House, Myung H. Yun, Chang S. Nam

**Affiliations:** ^1^Edward P. Fitts Department of Industrial and Systems Engineering, North Carolina State University, Raleigh, NC, United States; ^2^Department of Psychology, North Carolina State University, Raleigh, NC, United States; ^3^Department of Industrial Engineering, Seoul National University, Seoul, South Korea

**Keywords:** ACT-R, EEG, neural correlates, Granger causality, effective connectivity, multitasking, cognitive modeling

## Abstract

This study investigated the effect of task demand transitions at multiple levels of analysis including behavioral performance, subjective rating, and brain effective connectivity, while comparing human data to Adaptive Control of Thought-Rational (ACT-R) simulated data. Three stages of task demand were designed and performed sequentially (Low-High-Low) during AF-MATB tasks, and the differences in neural connectivity during workload transition were identified. The NASA Task Load Index (NASA-TLX) and the Instantaneous Self-Assessment (ISA) were used to measure the subjective mental workload that accompanies the hysteresis effect in the task demand transitions. The results found significant hysteresis effects on performance and various brain network measures such as outflow of the prefrontal cortex and connectivity magnitude. These findings would assist in clarifying the direction and strength of the Granger Causality under demand transitions. As a result, these findings involving the neural mechanisms of hysteresis effects in multitasking environments may be utilized in applications of neuroergonomics research. The ability to compare data derived from human participants to data gathered by the ACT-R model allows researchers to better account for hysteresis effects in neuro-cognitive models in the future.

## Introduction

In society today, people are inundated with situations in which their cognitive capacity is tested by the need or want to perform well in multiple tasks all at once. A common example comes in the form of piloting an aircraft. While flying, a pilot is tasked with watching through windshield for obstructions, weather conditions, and runway conditions while also monitoring airspeed, elevation, weather, fuel levels, and navigation information using monitoring equipment. At the same time, pilots must listen for auditory cues both from air traffic controllers as well as alarms such as ground proximity warnings. Pilots are also often tasked with manually guiding the plane using a steering mechanism as well as controlling acceleration manually. The level of automation may change given the sophistication of the technology in individual airplanes, but within any flight system, multitasking is required of the pilot.

Researchers have attempted to explain how behavioral and cognitive performance are impacted in such multitasking environments, and computer models have been developed to help simulate human performance and cognitive activity as well. Recently, increasing attention has been given to researching the impact of sudden shifts in task-demand in such environments on human performance and cognitive activity. The effect of a previous condition on a current condition, or hysteresis, is often the focus of such research. In the aforementioned aircraft pilot example, demand transitions may occur at multiple points during a flight depending on travel conditions as well as the need to perform a specific maneuver or procedure such as take-off or landing. Performing any of the given tasks poorly may have dire consequences not only for the pilot but also for any other crew, possible passengers, and cargo. If we understand the factors that may impact a pilot’s performance, which may include demand transitions, we can develop methods for counteracting such influencing factors if the impact is negative or promoting such factors if the impact is positive.

The main goal of the present research was to help explain not only brain region activation during such changes in task-demand transitions in a multitasking environment, but also a causal flow of cognitive functioning during and after such transitions. EEG data with performance data and subjective responses to the multitasking environment were analyzed. Additionally, we compared an established computer-based model for cognitive and behavioral performance with actual human performance. Specifically, this approach can search for discrepancies between human data and model data that may arise due to a lack of accounting for hysteresis effects within the computer-based model.

### Workload Transition and Hysteresis Effect During Multitasking

A hysteresis effect is described as the impact of previous demand conditions on current demand conditions (Morgan and Hancock, 2011). In workload transition research, this describes any impact that a previous level of workload, or task demand, may have on a new level of workload post transition. Previous research has sought to investigate the impact of hysteresis using a research design in which participants engage in a task (often a dual- or multitasking environment) during which there are changes in task demand. These studies often ask the participant to engage in a task that at some point will become significantly more or less demanding. While the order of levels of the task-demand presented to the participants may vary between studies, much of the existing literature currently available observes a hysteresis effect most commonly when an operator moves from a high to a low workload condition (Cox-Fuenzalida, 2007). Performance data is collected across time throughout the experimental task, with the researchers placing an emphasis on observable differences in performance between levels of task-demand as a measure of hysteresis.

Previous studies also often gather subjective ratings of mental workload from participants throughout the given experimental task, and approach changes in mental workload that correspond with changes in task demand as possible products of hysteresis. A high-demand or low-demand baseline is commonly used for comparison. The assumption is that if an individual suddenly changes from a high-demand task to a low-demand task, and the performance and/or mental workload are significantly different than a baseline performance and/or mental workload in the low-demand task, it is inferred that the high-demand task that occurred before the low-demand task had an impact on performance and mental workload during that low-demand task. Cox-Fuenzalida (2007) used the Bakan (1959) to observe the effects of transitions in task demand, and found that a significant increase in performance occurs when individuals transitioned from high to low task demand, but not from low to high task demand. Jansen et al. (2016) observed performance in a driving simulation task which was manipulated to define high- and low-demand conditions. Former research has demonstrated that during demand transitions, changes in mental workload often occur along with changes in performance. They also showed that subjective mental workload can remain elevated after switching from a high-demand task to a low-demand task (Jansen et al., 2016).

More recently, attention has been drawn to investigate the underlying relationship between the hysteresis effect and brain activity that is caused by the change of subjective mental workload. Schweizer et al. (2013) used fMRI to identify differences in brain activation by the level of workloads in distracted driving. Deprez et al. (2013) found differences in brain activation when single, dual, and multiple tasks in visual and auditory domains occur. Bowers et al. (2014) reported a presence of the hysteresis effect in gamma activity following a high- to low-demand transition as observed in a decreasing speed of gamma power. Several studies have described how workload transition effects, or hysteresis, develop over time by comparing multiple periods of aggregated performance data (e.g., Matthews, 1986; Gluckman et al., 1993; Ungar, 2005; Cox-Fuenzalida, 2007). Various cognitive models and approaches have been utilized to measure user performance in multitasking. Nevertheless, studies in the area of multitasking still have significant gaps: (1) only a limited number of the studies have quantitatively analyzed workload with temporal dynamics; (2) even fewer studies have shown how to predict and control user performance and cognition through a quantitative method including neurophysiological matrices; and (3) without a theoretical framework for workload transition effects, generalizing observations from one applied task to another applied task can be difficult (McKendrick, 2016). Therefore, it is necessary to develop a computational model to quantitatively analyze workload transition effects and multitasking performance.

Adaptive Control of Thought-Rational (ACT-R) is a high-level computational simulation of human cognitive processing and one of the cognition theories that seeks to predict human performance in multitasking. In this study, ACT-R was used as a theoretical framework to resolve the discrepancies seen in previous research, help us better understand the underlying physical and cognitive processes that occur within workload transitions, and predict the impacts of hysteresis effects. The ACT-R may be a useful tool for unifying multiple disciplines by incorporating neurophysiological data, behavioral data, and cognitive data into one model.

### Cognitive Architecture: ACT-R

Adaptive Control of Thought-Rational is a cognitive architecture that allows a high-level computational simulation of human cognitive processing (Anderson, 2007). Cognitive architecture refers to both a unified theory of cognition - the outline of the structure for various parts of the mind - and a computational implementation of the theory with specified rules and associative networks. The symbiotic relationship between cognitive modeling and cognitive neuroscience results in palpable progress toward the shared goal of better understanding the functional architecture of human cognition. Specifically, cognitive architectures are frameworks that can be used to develop computational models of human cognitive processes (Langley et al., 2009; Taatgen and Anderson, 2010; Smart et al., 2016). Computational cognitive models such as ACT-R are formal theories of cognition that provide predictions of human performance in cognitive tasks such as multitasking. These models generally include simulated representations of human cognitive processes such as memory, attention, visual and motor processing, problem solving, learning, and other related phenomena. Formal cognitive models for predicting, capturing, and understanding multitasking as a manifestation of underlying cognitive processes are built from these cognitive architectures. ACT-R, principally developed at Carnegie-Mellon University by John Anderson, was created to help mechanistically demonstrate human reasoning and memory faculties (Whitehill, 2013). It does so by describing how people recall “chunks” of information from memory and how they solve problems using “production rules” in order to break down goals into sub-goals. ACT-R is a rule-based system that has been widely used by cognitive scientists to model human cognitive performance. It is also one of the few cognitive architectures that has an explicit link to research in the neurocognitive domain: the structural elements of the core ACT-R architecture (i.e., its buffers and modules) link with different regions of the human brain (Anderson, 2007). This enables cognitive modelers to predict the activity of different brain regions at specific junctures in a cognitive task (Anderson, 2007). ACT-R is a formalized, integrated cognitive architecture that combines the Spreading Activation Memory theory with a production system to model the high level of cognitive tasks. Like many successful architectures, ACT-R is a modular theory that aims to provide an integrated account of human cognition.

**FIGURE 1:**
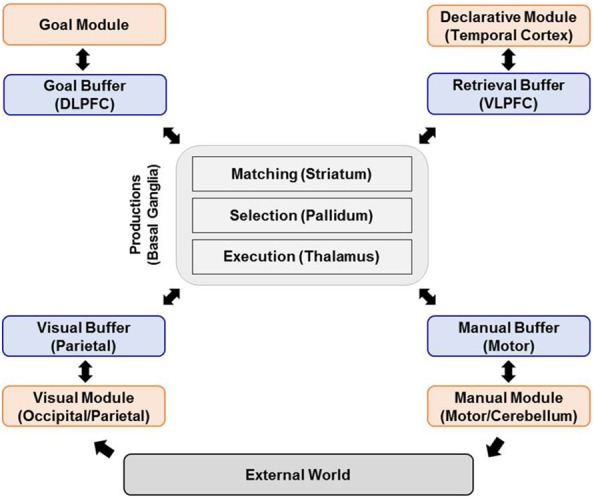
Overview of ACT-R modules (revised based on Anderson et al., 2004).

Figure 1 shows an overview of ACT-R modules (Anderson et al., 2004). It treats the mind as being composed of distinct modules based on functionality. The ACT-R modules represent the functions of the brain as well as how these functions are mapped to different parts of the brain. ACT-R incorporates both declarative knowledge (e.g., addition facts) and procedural knowledge (e.g., rules for solving multi-column addition) into a production system where procedural rules act on declarative chunks (Anderson et al., 2004). In ACT-R, the structures of declarative knowledge are called “chunks” and are held in the Declarative module, whereas those of procedural knowledge are called “rules” and are held in the Procedural module. The rules also have access to other modules, including the Visual module for perception, the Manual module for action, the Imaginal module for storing visual problem representation, and the Goal module for keeping track of current intentions. These modules are linked to specific areas of the brain: Manual in the motor cortex (BA 3/4), Imaginal in the parietal cortex (BA 39/40), Declarative in the dorsolateral prefrontal cortex (DLPFC) (BA 45/46), Goal in the anterior cingulate cortex (ACC) (BA 24/32), Visual in the fusiform gyrus (BA 37), and Procedural in the caudate of the basal ganglia (Matessa, 2008). Each module of ACT-R has its own buffer that can store only one chunk of information extracted from the corresponding module. In ACT-R, the condition statements of all production rules are compared with the current contents of buffers every 50 ms (Anderson, 2007).

When there are one or more matching productions, the matching one or the one with the highest expected value among them, respectively, is selected to fire, and the action statement of the selected rule then makes each module exchange chunks of information or creates new chunks in respective modules (Taatgen and Lee, 2003). At this time, only one chunk can be processed through each buffer at a time, while all other modules can be executed simultaneously. For example, the declarative module cannot retrieve the additional memory chunk until it has completed the previous retrieval. In contrast, the manual module can perform the physical movement, while the visual module looks at a new object. Moreover, there is a limit to the number of objects and a time limit in the visual and retrieval buffer. These are related to the working memory limitation of human cognitive processing (Anderson et al., 2004). ACT-R has a sub-symbolic level in which continuously varying quantities are processed in parallel to produce much of the qualitative structure of human cognition. These sub-symbolic mechanisms contribute to representing neural-like activation processes that determine both the speed and success of access to chunks in declarative memory as well as the conflict resolution among production rules (Anderson et al., 2004). The core of ACT-R memory modeling can be summarized with the following equations (see Table 1).

**Table 1:** Sub-symbolic mechanisms in ACT-R.

Mechanism	Equation	Description
Activation	$\text{A}\left(\text{t}\right)\;=\;\text{B}\left(\text{t}\right)+\sum_{j}W_{\text{j}}{\text{S}}_{\text{ij}}+\text{ε}$	B(t): base-level activation S_ij_: strength of association of element j to chunk i *W_j_:* attentional weighting of element j of chunk i 𝜀: Noise level
Base-level learning	$B(t)=\ln\sum_{k}{\text{t}}_{\text{K}}^{\text{-d}}\;\text{+}\;\text{B}$	*k*: the number of experiences for chunk d: decay rate for the event k t_k_: time of the event k
Retrieval probability	$p_{i}=\frac{x-\text{μ}}{1+e\frac{\text{τ}-A_{i}}{S}}$	τ: retrieval activation threshold *s*: amount of noise in the system *A*_i_: expected activation
Time of retrieval	*Time = Fe^-A^*	*A*: the activation of the chunk which is retrieved *F*: the latency factor parameter


A(t) refers to the activation of a particular chunk and B(t) is the base activation of the knowledge chunk at time t, and the summation is the associative strength of the chunk, defined as “the elements in the current goal chunk and the elements currently being processed in the perceptual field” (Anderson, 1983, p 51). The base activation B(t) is increased whenever it is used, either through practice (learning events) or when matched to a production rule. If we retrieve memory and there is a matching memory chunk, that chunk will be recalled when the retrieval activation threshold, *τ* is exceeded. The probability of retrieval depends on the expected activation, A_i_, and the amount of noise, *s*. The *s* parameter represents the sensitivity of recall to changes in activation. When a retrieval request is made, the time required for the chunk retrieval is available in the retrieval buffer. ***A*** indicates the activation of the chunk which is retrieved, and ***F*** represent the latency factor parameter. These equations provide an integrated foundation for theory development in memory encoding, storage, and retrieval.

### ACT-R and Neuroimaging Studies

By comparing simulated human cognitive data from the ACT-R architecture to real human data, the cognitive mechanisms behind multitasking and the effects of changes in internal cognitive strategies, external interface properties, and task demands on brain networks yield important insights. Research using Functional Magnetic Resonance Imaging (fMRI) has identified the roles of various brain regions and assigned the cognitive functionality of those regions to ACT-R modules and buffers (Cassenti et al., 2011). Despite the strong correlation between fMRI data and ACT-R, it still remains unknown whether ACT-R also holds a strong correlation with Electroencephalogram (EEG) data. This has recently piqued interest among researchers who have been outlining methods to study the neural correlates of ACT-R in electrophysiological data and demonstrating how different ACT-R modules can be associated with observable EEG data (Cassenti et al., 2011; van Vugt, 2012, 2014). Despite initial attempts to correlate ACT-R and EEG data, more investigation is still required. As a result, this study aimed to extend these initial findings by further exploring EEG correlates of ACT-R modules and discuss the broader implications of this approach for both cognitive neuroscience and cognitive modeling with ACT-R. Specifically, this study developed models of multitasking behavior in a realistically complex workspace by incorporating a wide range of cognitive processes that are affected by task demand transitions. Multitasking environments can often give rise to such transitions in task demand and subjective mental workload, as the operator is actively monitoring and engaging in several different tasks at once. This study extended the previous findings by manipulating task demand (Low-High-Low) and observing changes in brain connectivity across the demands in a multitasking setting to improve operators’ cognition by comparing ACT-R simulated data. Surprisingly, however, little is known about causal relationships between ACT-R modules and matched brain areas activated during task demand transitions. In particular, brain effective connectivity using Granger Causality (GC) has gained a great deal of attention recently. GC is a method for investigating whether one time-series correctly predicts another and allows us to analyze brain circuit connections and how they change over the course of a cognitive process (Coben and Mohammad-Rezazadeh, 2015). This analysis provides a means to study time-varying interactions between brain areas and cognitive architecture components (Seth et al., 2015). After careful systematic review, however, no prior studies have assessed changes in GC influence in the context of demand transitions.

The purpose of this study was to investigate the changes in neural connectivity during a hysteresis effect that may accompany task demand transitions using GC. Two levels of task demand, including a low-demand period and a high-demand period were used to identify differences in neural connectivity during transitions between the levels. The NASA Task Load Index (NASA-TLX) and the Instantaneous Self-Assessment (ISA) were used to measure the subjective mental workload during the different demand levels giving us the ability to draw comparisons between mental workload at a given level of task demand and previous levels of task demand. In addition, based on observed behavioral and neural data, the activation of ACT-R modules were used to clarify the empirical correspondence between human data and computer cognitive modeling. To better understand this process, a computational model of multitasking was developed, and its performance was juxtaposed against human operators performing the same task.

## Materials and Methods

### Participants

A total of twenty participants (14 male; 6 female) recruited from a local university participated in the present study. Participants were given monetary compensation for their participation. All participants successfully completed the entire experiment and were included in the data analyses. Participant mean age was 26.7 years (standard deviation, *SD* = 4.37). Participants reported being free of any medical or neurological disorders and had normal or corrected vision. Participants gave their written consent after a detailed explanation of the experimental procedure which was reviewed and approved by the University’s Institutional Review Board. All participants had no experience with the AF-MATB system before this training, so the proficiency level before training was assumed to be the same for all participants.

### Stimuli and Experimental Task

**FIGURE 2:**
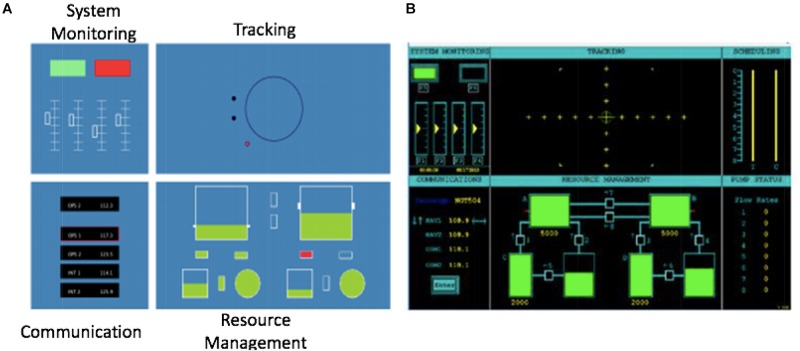
Experimental task interface **(A)** mMATB **(B)** AF-MATB. AF-MATB is a research paradigm where participants perform a tracking task while concurrently monitoring warning lights and dials, responding to computer-generated auditory requests to adjust radio frequencies, and managing simulated fuel flow rates using various key presses.

Participants were trained on the Air Force Multi-Attribute Task Battery (AF-MATB, Miller, 2010; see Figure 2), a computer-based multitasking environment designed to evaluate operator performance and mental workload while human operators perform a benchmark set of tasks similar to activities that aircraft crew members perform in flight (e.g., system monitoring, resource management, communications, and tracking tasks) (Comstock and Arnegard, 1992).

The Multi-Attribute Task Battery (MATB) was developed by the National Aeronautics and Space Administration (NASA) to evaluate human performance in a multitasking environment. The United States Air Force version of MATB (AF-MATB) operating in information throughput (IT) mode was implemented in this study (Bowers et al., 2014). AF-MATB requires the human operator to simultaneously monitor and respond to four independent tasks on one computer screen. The tasks consist of Systems Monitoring, Communication, Targeting, and Resource Management. For this study, each of the four tasks were equally weighted, so no task had greater importance than another task. The following depicts the objectives for each task: The System Monitoring task is in the top left corner of the MATB window and consisted of two subtasks: lights and dials. The two rectangles at the top represent the lights. The participant is asked to keep the left light in the on status “displaying green” and the right light in the off status “displaying black.” If the lights switched from these initial conditions, selecting F5 or F6 keys reset the lights. Beneath the lights are four vertical columns which represent dials. Throughout the task, yellow markers within the dials continuously oscillated between one location above and below the center of the dial. Occasionally, the yellow marker shifted toward the top or the bottom of the dial and began oscillating around a new location. When this event occurred, participants were to select the corresponding F1–F4 keys to reset the dials.

The Communication task is in the bottom left corner of the MATB window. The objective of the communication task was to alter the channel and frequency with audio cueing. An audible message instructed the participants to modify a specific communication channel to a given frequency. The participants navigated to the appropriate channel and set the frequency by selecting the up, down, left, and right arrow keys. For example, the participant had to discriminate his or her callsign (e.g., “NGT504”) from other audio messages for different callsigns. These extraneous, auditory cues can be thought of as distractors.

The Targeting task is in the top right corner of the MATB window. Throughout the task, the green cursor drifted around the window. The objective was to maintain the green cursor within a larger yellow circle found in the center of the window by using a joystick. The Resource Management task is in the bottom right corner of the MATB window. The objective of the Resource Management task was to maintain a fluid level within a specific range in two primary tanks. This was accomplished by turning “on” and “off” four reservoir tanks by using number keys. It is important to note that the fluid level is continuously flowing, and therefore must be monitored regularly. Flow is sometimes hindered, as flow between tanks can be disrupted without input by the participant, forcing the participant to adjust flow between the tanks.

### Experimental Procedure

Through pre-experimentation surveying, demographic information was collected, and any previous subject experience with AF-MATB or a similar system was noted. Following the survey session, participants proceeded through a training session in order to become familiarized with the experiment tasks (AF-MATB).

To ensure that all participants had a similar level of performance, Bowers (2013)’s recommendations was employed, and training was repeated until they reached a criterion at a level of approximately 65% correct response on average in the System Monitoring and Communications tasks. In this study, on average training lasted for 15 ± 3 min (6 ± 2 trials) on the System Monitoring and Communications tasks. In addition to practicing these AF-MATB trials, the participants were also introduced to the NASA TLX and the ISA rating since both measures were presented during data collection. Participants read the instructions associated with each measure and practiced filling out the forms.

**FIGURE 3:**
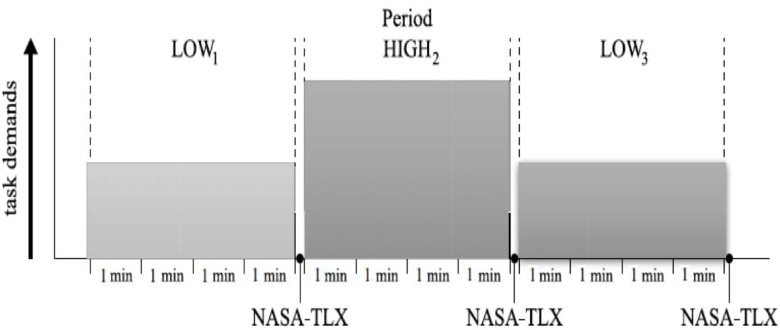
Experimental progression for one session. The main experiment was composed of four sessions, each session having three experimental conditions with a low-high-low demand schedule. Each condition lasted 4 min, and participants were required to perform four tasks simultaneously during each trial. Participants completed the NASA-TLX questionnaire after each condition.

No participants failed to reach this criterion or had to repeat additional training trials. Afterward participants performed the main experimental tasks. The main experiment was composed of four sessions, each session having three experimental conditions with a low-high-low demand schedule (see Figure 3). Each condition lasted 4 min and participants were required to perform four tasks simultaneously during each trial. Participants completed the NASA-TLX questionnaire after each condition. Performance in each subtask is individually scored. All participants were tested individually in a laboratory and the experiment took approximately 1.5 h for each participant.

#### ACT-R Modeling

**FIGURE 4:**
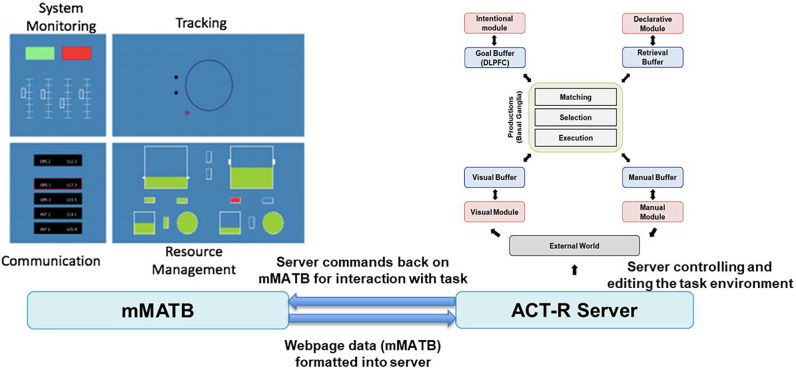
SIMCog-JS Framework: mMATB (revised based on Halverson et al., 2015). SIMCog-JS (Simplified Interfacing for Modeling Cognition – JavaScript) allows models to interact with browser-based software, and it specifies how elements in the interface are translated into ACT-R chunks. The software allows joystick and keyboard interaction with JavaScript code, and it sends ACT-R commands from the external software.

Adaptive Control of Thought-Rational data were recorded within a JavaScript implementation of a modified Multi-Attribute Task Battery (mMATB-JS, Halverson et al., 2015; see Figure 4). This web-based version of the MATB contains up to four simultaneous tasks that are the same as the human-operated Air Force Multi-Attribute Task Battery (AF-MATB, Miller, 2010). In the mMATB-JS, all task parameters were matched to AF-MATB setting. Task demands were manipulated by increasing the event rate in each of the subtasks. Three demand conditions were implemented in a fixed order of Low-High-Low. The output of model simulations included behavioral data like that produced in human observations, such as response time and error rate. Over the course of a mMATB session, the time series data for joystick movement on the screen and various button presses were recorded, and the utility of multitasking behavior when comparing human-to-model data was examined. In order to compare the human performance data to the ACT-R model, Independent Components (ICs) derived from EEG data were linked to ACT-R buffer activation using dipole fitting and brain effective connectivity analysis. Prezenski and Russwinkel (2016) demonstrated that EEG data could be used to validate ACT-R models. If the ICs match specific ACT-R modules, the timing of peaks of buffer activity should match IC-peaks. In this study, both IC-peaks timing and brain connectivity between ICs were observed. In the ACT-R model, the modules are designed to occur in specific areas of the brain: Imaginal in the parietal cortex, Declarative in the dorsal lateral prefrontal cortex (DLPFC), Goal in the anterior cingulate cortex (ACC) and Visual in the fusiform gyrus (Borst et al., 2010).

#### EEG Acquisition and Pre-processing

Electroencephalogram signals were recorded using an EEG cap (Electro-Cap International, Inc.) embedded with 62 active electrodes covering frontal, central, parietal and occipital areas and were based on the modified 10–20 system of the International Federation (Sharbrough et al., 1991). Recordings were referenced to the left ear lobe and grounded between AFz and Fpz. EEG signals were amplified with a g.USBamp amplifier (g.tec Medical Engineering). EEG signals were sampled at 512 Hz, with band-pass filtered between 0.01 and 75 Hz to take out unwanted frequency bands, and notch-filtered at 60 Hz.

To identify and remove artifacts, all trials were inspected in three main ways. First, the reject function in EEGLAB (Delorme et al., 2011) screened out high-noise trials based on the kurtosis of each trial. Second, each trial was then manually inspected to exclude trials that contained electrode drift noise and muscle movement-related noise. Finally, independent component analysis (ICA) was used to decompose the EEG signal into independent components (ICs). All ICs were visually inspected, and components that resembled EOG activity were rejected from further analysis. An average of three trials were rejected for each subject. Signal acquisition and processing were all conducted using BCI2000 system (Schalk et al., 2004), MATLAB (The MathWorks), and EEGLAB (Delorme et al., 2011).

### Experimental Design and Independent Variables

Task demands were manipulated by increasing the event rate in each of the subtasks (see Table 2) based on the previous study (Bowers et al., 2014). The event rate ranges used for our study were taken from prior work (Bowers, 2013) which classified event rates as easy (low) or hard (high) based on behavioral performance for various event rate settings. The difficult levels of the tracking task, however, were set to default settings for the following reasons. First, the level of difficulty is predetermined by the number of directional changes of the reticle and the speed at which the reticle moves, and second, the current AF-MATB system does not allow to change them.

**Table 2:** Rate at which events occur in each level of task difficulty.

			Task demand: event rate (per minute)	
			Low	High
Subtasks	Communications	Target	0.7∼1.0	3∼4.2
		Distractor	0.35∼0.5	1.0∼1.35
	System	Lights	4.0 ∼ 6.7	15∼17.7
	monitoring	Gauges	5.0∼7.0	16∼18
	Resource	Failures	0.7∼1.0	4.3∼5
	management	Shut-offs	0.7∼1.0	2∼2.3


Three experimental conditions were used with a fixed order: LOW1 (L1), HIGH2 (H2), and LOW3 (L3). Hysteresis was examined by comparing performance and mental workload in L3 with L1.

### Dependent Variables and Data Processing

#### Behavioral Data

Within the AF-MATB system, there are several performance measures. The average correct reaction time was analyzed for the System Monitoring task and the Root Mean square (RMS) was analyzed for the Tracking task, as the use of these performance measures in workload transition studies was indicated in previous research (Bowers et al., 2014). RMS is the distance between the moving cursor and the center point of the Tracking task. While behavioral data was also collected for the communication task and the resource management task, event rates for these tasks did not provide enough data to allow for meaningful interpretation of a 2-min time bin.

#### Subjective Rating

Two measures of mental workload were taken, namely, NASA-TLX and ISA. The NASA Task Load Index (TLX) (Hart and Staveland, 1988) includes six components: mental demand (MD), physical demand (PD), temporal demand (TD), the individual’s perceived level of performance (PE), effort (EF), and frustration (FR). The unidimensional Instantaneous Self-Assessment (ISA) scale (Tattersall et al., 2007) was used to assess mental workload during experimental conditions. The ISA item (i.e., “Report how much mental workload the task just required”) was collected verbally two times during each condition. A 7-point Likert scale was used, where 1 corresponded with a very low mental workload and 7 with a very high mental workload.

### Neurophysiological Data

#### Effective Connectivity Analysis

**FIGURE 5:**
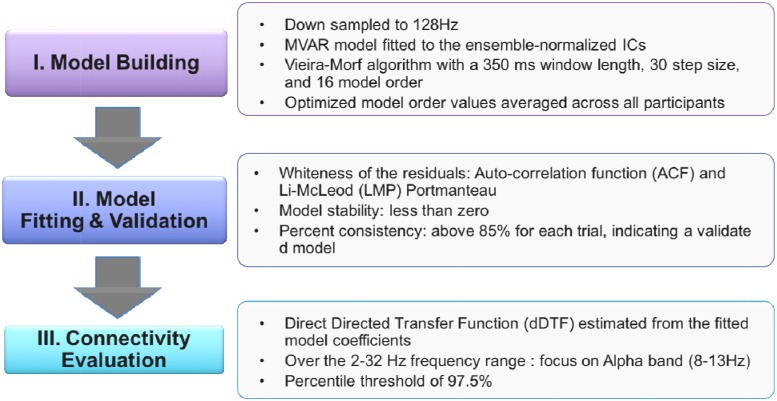
Connectivity analysis procedures.

For effective connectivity analysis (see Figure 5), data was down-sampled to 128 Hz. Following Independent Component Analysis (ICA) and artifact rejection procedures, all retained ICs were localized using DIPFIT. The Source Information Flow Toolbox (SIFT) for EEGLAB was used to evaluate effective connectivity, the causal flow of information between brain sources (Delorme et al., 2011).

Given k EEG time series *X* (*t*) = [*x*_1_ (*t*) × *x*_2_ (*t*)…*x*_k_ (*t*)], the multivariate vector autoregressive (MVAR) of order p is $X\left(t\right)=\sum_{n=1}^{\text{p}}A\left(n\right)X\left(t-n\right)+E\left(t\right),$ where A(n) is the coefficient of the model as shown below and E(t) is the model error.

$$A\left(n\right)=\left[\begin{array}{ccc} a_{11}^{\left(n\right)} & \cdots & a_{\text{1k}}^{\left(n\right)}\\ \vdots & \ddots & \vdots \\ a_{\text{k1}}^{\left(n\right)} & \cdots & a_{\text{kk}}^{\left(n\right)} \end{array}\right]$$

Multivariate Granger causality between the time series can be inferred from the model coefficients (Kamiñski et al., 2001). The estimation of the coefficients can also be achieved in the frequency domain. This gives rise to several metrics including the directed transfer function and partial directed coherence (Kuś et al., 2004). In this study, a multivariate autoregressive model (MVAR) was fit to the ensemble-normalized ICs using the Vieira-Morf algorithm with a 350 ms window length, 30 step size, and 16 model order. Model order was optimized from 1 to 40 such that the Hannan-Quinn criterion for each participant was minimized (Kim et al., 2017). Then the optimized model order values were averaged across all participants.

To validate the MVAR model, the whiteness of the residuals, model stability, and percent consistency were determined for each trial. The auto-correlation function (ACF) and the Li-McLeod Portmanteau (LMP) tests were used as whiteness test criteria. The LMP test was used for its improved small-sample properties and lack of variance inflation compared to other available Portmanteau tests (Mullen, 2010). In addition to meeting the ACF and LMP criterion, the model stability was less than zero, and the percent consistency was above 85% for each trial indicating sufficient model validation.

Following model fitting and validation, SIFT was used to evaluate connectivity. The direct Directed Transfer Function (dDTF), a measure of frequency-domain conditional Granger causality, was estimated from the fitted model coefficients. The Directed Transfer Function (DTF) allows for analysis of short epochs of EEG activity to analyze information flow between different brain structures while also making it possible to determine spectral content of the signal (Kamiñski et al., 2001). However, DTF is limited by its ability to differentiate between direct and indirect connections. By combining DTF and partial coherence measures, dDTF quantifies conditional, directionally specific information transfer between sources over the trial time period at each frequency (Korzeniewska et al., 2003). In this study, dDTF was determined over the 2–32 Hz frequency range. A percentile threshold of 97.5% was used for each frequency to visualize relevant directional connections between brain sources.

### Statistical Analysis

The 3 (condition; L1, H2, L3) × 6 (2 min = 1 time bin) repeated measures ANOVAs for each performance measure were followed up with one-way ANOVAs to compare performance and mental workload within each 2 min interval as a variation of workload condition. For the measures with significant main effects in difficulty condition in this comparison, Tukey’s Honestly Significant Difference (HSD) tests were used to determine which conditions had significantly different means. The results also include partial eta squared ($\eta_{p}^{2}$) to measure the effect sizes as well as the percentage of total variability associated with an effect (Cohen, 1992).

## Results

### Behavioral Data

Overall, the results showed significant differences between conditions Low and High workload across the 6 time bins (see Table 3).

**Table 3:** Mean and standard deviation of behavioral data by workload condition.

Level of difficulty			Low1		High2		Low3	
Timeline (min)			0–2	2–4	4–6	6–8	8–10	10–12
System monitoring	Correct RT (s)	Human	1.69 (0.46)	1.85 (0.56)	1.85 (0.54)	1.98 (0.32)	1.38 (0.18)	1.51 (0.31)
		ACT-R	1.58 (0.23)	1.72 (0.22)	1.88 (0.36)	1.92 (0.42)	1.59 (0.35)	1.61 (0.22)
Tracking	RMS (%)	Human	45.83 (11.4)	46.92 (12.2)	77.01 (15.3)	84.32 (17.8)	43.62 (13.5)	42.78 (14.3)
		ACT-R	43.72 (9.20)	45.19 (10.3)	76.23 (14.8)	80.29 (12.4)	45.29 (10.7)	44.64 (13.2)


A significant effect of difficulty level on correct reaction time for the system monitoring task was found: *F*(2, 141) = 27.46, *p* < 0.001, $\eta_{p}^{2}$ = 0.238. The tracking RMS error rate also showed a significant effect in level of difficulty, *F*(2, 141) = 102.41, *p* < 0.001, $\eta_{p}^{2}$ = 0.217. To further investigate those effects, Tukey’s HSD tests were used to compare performance averages from the six time bins. The tests evaluating system monitoring reaction time indicated that the L3 condition had a significantly shorter reaction time than the L1 condition (*p* < 0.01) despite the absolute difficulty level being the same. Furthermore, the reaction time between the second time bin (2–4 min) with the L1 condition and the fifth time bin (8–10 min) with the L3 condition were significantly different (*p* < 0.01). These findings imply that there was a hysteresis effect caused by demand transitions in performance during multitasking. Finally, there was no significant difference between L1 and L3 on RMS error rate.

### Subjective Mental Workload

We found a significant effect for level of difficulty on ISA rating and NASA TXL total workload score: *F*(2,141) = 124.95, *p* < 0.001 and *F*(2, 141) = 53.43, *p* < 0.001, respectively (see Table 4). Thus, the manipulations of AF-MATB task difficulty proved to be reflected in performance and mental workload across the time frame of an experimental condition (see Table 3). Additionally, time bins had a significant effect on reaction time and RMS error rate: *F*(5,138) = 12.54, *p* < 0.001 and *F*(5,138) = 43.20, *p* < 0.001, respectively, and the effect of time bins on ISA rating was also found to be significant: *F*(5,138) = 50.93, *p* < 0.001.

**Table 4:** Mean and standard deviation of subjective mental workload by workload condition.

Level of difficulty	Low1		High2		Low3	
Timeline (min)	0–2	2–4	4–6	6–8	8–10	10–12
ISA rating	3.11	3.27	5.33	6.11	2.94	2.72
	(1.07)	(1.22)	(0.84)	(0.75)	(0.85)	(0.10)
NASA TLX	56.868		76.542		51.56	
Total workload	(12.30)		(8.27)		(13.20)	


### Behavioral Data and ACT-R Model Data

**FIGURE 6:**
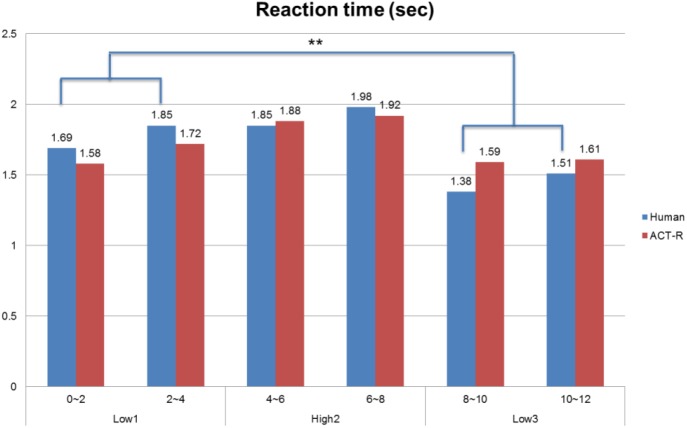
Comparison between human behavioral data and ACT-R model outcomes for reaction time of the system monitoring task (^∗∗^*p* < 0.05).

The manipulations of AF-MATB task difficulty proved to be reflected in human performance data. As mentioned earlier, the Tukey’s HSD tests evaluating system monitoring reaction time indicated that the L3 condition had a significantly shorter reaction time than the L1 condition despite the absolute difficulty level being the same (see Figure 6). For the ACT-R simulation data, however, there was no significant difference between the L1 and L3 condition even though the other measures showed overall good fits with the human data.

### Neurophysiological Data

#### Effective Connectivity Data and ACT-R Model Data

The data was common-average referenced and zero-phase, high-pass filtered at 0.1 Hz. The 2 min EEG epoch was imported in EEGLAB for the further preprocessing. After importing, the datasets were separated into maximally independent components using Infomax ICA (Bell and Sejnowski, 1995). These sources were localized using both a single or dual-symmetric equivalent-current dipole model and a four-shell spherical head model co-registered to the subjects’ electrode locations by warping the electrode locations to the model head sphere. Table 5 lists the cortical regions associated with Brodmann’s areas (BAs) localized across the conditions.

**Table 5:** Localized cortical sources and associated Brodmann’s area (BA) for each condition.

Difficulty level	Cortical sources (BA area)
Low 1	BA(17): visual cortex (VC)
	BA(32): dorsal anterior cingulate (dACC)
	BA(31): dorsal posterior cingulate (dPCC)
	BA(24): ventral anterior cingulate (vACC)
High2	BA(7): parietal cortex (PC)
	BA(10): prefrontal cortex (PFC)
	BA(17): visual cortex (VC)
	BA(32): dorsal anterior cingulate (dACC)
	BA(39): parietal cortex (PC)
Low3	BA(10): prefrontal cortex (PFC)
	BA(17): visual cortex (VC)
	BA(24): ventral anterior cingulate (vACC)
	BA(31) : dorsal posterior cingulate (dPCC)


In order to compare human performance and the ACT-R model, Independent Components (ICs) derived from EEG-data were linked to ACT-R buffer activation using dipole fitting and brain effective connectivity analysis. This was used to examine the utility of behavior in a multitasking environment when comparing human data to model data. Once the electrophysiological correlates of ACT-R were determined by goodness of fit, the interaction between multiple modules within the ACT-R model could be monitored by analyzing patterns of synchronization in brain network.

#### Brain Network Analysis

The mean causal information flow, as measured by the dDTF, was analyzed between the localized cortical sources at each task difficulty level at the alpha and beta bands (7–30 Hz). The frequency band was based on Time-Frequency causal flow analysis.

Table 6 shows transient information flow during the AF-MATB task at three conditions across the whole trial. The neural network is modeled by several nodes that each represent a single brain region and corresponding connected edges that represent the interaction between each brain region. The color of the edges represents connectivity strength (i.e., the amount of information flow along that edge). A red edge represents connectivity while green represents low connectivity. The width of the edge represents connectivity magnitude (absolute value of connectivity strength, [min max] = [0.001 0.1178]). The color of the node represents the asymmetry ratio of connectivity for that source. The asymmetry ratio indicates whether all connectivity related to that node is inflowing or outflowing. It ranges from -1 to 1. A red color (close to +1) indicates that a node is causal source, blue (close to -1) means that a node has a role of causal sink, and green (close to 0) represents a balanced flow. The size of a node represents the amount of information outflow ([min max] = [0.001 0.2232]) from the source. In Table 6, figures in the second column show the changes in causality flow over the task. For the first L1 condition, the visual cortex and ventral cingulate cortex node are colored red and yellow, respectively. This indicates that these two components are the causal source for the whole network. The edge width and color between the visual cortex (VC) and dorsal anterior cingulate cortex (dACC) coupling have a sky-blue hue and are thicker than the others. Secondly, under the H2 condition, the causality flow was observed between the parietal cortex (PC) and dorsal anterior cingulate cortex (dACC). The node color of dACC and PFC are red and yellow, respectively, which means those nodes would be a hub node for the high-demand condition.

**Table 6:** Effective connectivity and network metrics by workload conditions.

	Low1		High 2		Low 3	
	0 ∼ 2min	2min ∼ 4min	4min ∼ 6min	6min ∼ 8min	8min ∼ 10min	10min ∼ 12min
Brain network pattern	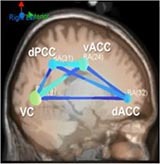	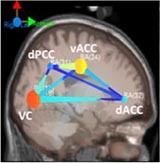	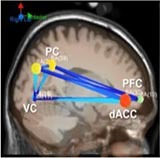	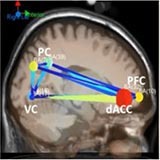	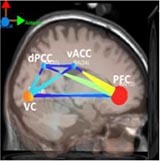	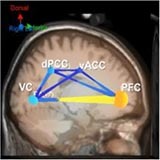
From–to	VC → vACC	vACC → dPCC	dACC → PFC		PFC → vACC	
Asymmetry ratio	0.167	0.214	0.683	0.789	0.713	0.496
	(0.011)	(0.032)	(0.026)	(0.042)	(0.012)	(0.052)
Outflow	0.079	0.163	0.197	0.218	0.203	0.178
	(0.003)	(0.045)	(0.052)	(0.061)	(0.021)	(0.043)
Connectivity magnitude	0.026	0.043	0.032	0.041	0.077	0.058
	(0.001)	(0.020)	(0.006)	(0.007)	(0.010)	(0.005)


## Discussion

This study investigated the effects of task demand transitions and the hysteresis effect that occurs at multiple levels of analysis (behavioral, subjective mental workload, and effective connectivity). In the performance data, there was a significant difference between the L1 condition and the L3 condition. In the System Monitoring task, correct reaction time was shorter after the High to Low demand transition. Mental workload ratings during the L3 condition are lower than the L1 condition which is consistent with previous findings (Hancock et al., 1995), even though the first and third trials were performed at an identical difficulty level. According to results from the effective connectivity analysis, the PFC was the hub node in the L3 condition unlike the L1 condition. Furthermore, this study suggests that the H2 demand condition, which represents activation of prefrontal cortex, has an impact on the L3 condition. This study was also able to compare hysteresis effects from human participants within a multitasking environment to the computer-based ACT-R model. This presents an opportunity for hysteresis effects to be incorporated in future cognitive modeling, furthering the breadth of the understanding for cognitive processing interpreted through EEG data.

### Behavioral and Neural Correlates of Hysteresis Effect

Under the low condition (L1), VC and vACC was the source node to dACC; however, under the L3 condition, the vACC area was a causal sink connected with the PFC. The core functions of the ACC are mainly divided into the error detection, task preparation, and emotion regulation (Nieuwenhuis et al., 2001). PFC is involved in complex cognitive behavior and decision making. This area monitors memorization and recognition function and contributes to the formulation of complex behavior based on collected information as compared to ACC (Goldman-Rakic, 1996; Kim et al., 2018). For this reason, the directional change can be explained as a hysteresis effect. In other words, after completing the high demand condition, the prefrontal area remained active, so the direction and magnitude of connectivity within the neural network might differ from the first demand conditions (L1). This presents a potential reason why participants reported lower mental workload during the third, low-demand conditions (L3).

### ACT-R Module Activation

For the L1 condition, strong information flows between the visual cortex (VC) and dorsal anterior cingulate cortex (dACC) were observed. Following the first transition of workload (Low-High), the causality flow was observed between the parietal cortex (PC) and dorsal anterior cingulate cortex (dACC) indicating a connection between imaginal modules and goal modules. Lastly, after the second workload transition (i.e., from High to Low), we observed a causal flow from prefrontal cortex to dACC indicating connectivity between declaratives modules and goal modules. By monitoring the interactions between modules, this study can improve the understanding of how different brain regions interact within the ACT-R model and presents a means to enhance existing cognitive models. Brain network analysis allows for the interpretation of causal relationships and efficiency across cognitive model components and specific brain regions. Future work is necessary to develop this multitasking model to account for applied workload transition effects and conduct further real-time analysis to integrate brain network dynamics and model development. Continued interdisciplinary research of cognitive modeling and neuroscience will lead to better understanding of cognitive processes of the human brain at work.

### Limitations and Future Research

Few limitations of the current research along with questions for future research should be noted. First, the sample size (*n* = 20) may not be large enough to generalize the results. It may not reflect the different workload transition situations regarding to different user groups’ performance and perception across diverse contexts. Thus, this study can only be used as a reference of Hysteresis effects on three stages of task demands performed sequentially (i.e., Low-High-Low). A larger sample size with a wide range of ages would increase the validity of the future studies. In addition, as a research tool to measure one’s mental workload, the NASA-TLX has at least two limitations: (i) it can be intrusive and disruptive to primary task performance, and (ii) workload estimates are based on an opinion not an objective measurement. This study employed the NASA-TLX to measure workload for a few reasons: its established reliability and validation, its proved usefulness in various multitasking contexts such as real (e.g., Shively et al., 1987) and simulated flight tasks (e.g., Nataupsky and Abbott, 1987; Battiste and Bortolussi, 1988; Vidulich and Bortolussi, 1988; Corwin et al., 1989; Tsang and Johnson, 1989), and its past use in studies of Hysteresis effects on mental workload (e.g., Hancock et al., 1995; Morgan and Hancock, 2011). Moreover, participants were given the NASA-TLX after one workload condition was completed and before the next condition began (e.g., between Low and High) which took about 1 min to complete. For this reason, it is unlikely that the administration of the NASA-TLX affected primary task performance. Lastly, this study did not use control demand transitions (e.g., Low-Low-Low; High-High-High) which could have served as a baseline to compare other experimental conditions. Since one of the main goals in the present study was to, first, assess neural correlates of the Hysteresis effect that has already identified and validated by other behavioral research and, second, model it using ACT-R, we did not investigate controlled demand transitions. Future research, however, may incorporate such control groups in order to re-confirm the hysteresis effect observed.

The limitations of this research promote a variety of future research directions. This study offered two main research topics that may help continue to build the strategies for modeling cognitive function and further our understanding of hysteresis. First, individual working memory capacity could be taken into consideration while gathering behavioral, subjective, and neurophysiological data. Baddeley (2012) defines Working Memory as the systems serve as keeping things in our mind while performing complex tasks such as reasoning, decision making and learning. Previous research has observed individual differences in Working Memory Capacity (e.g., Barrett et al., 2004). Since multitasking environments require the recollection of multiple tasks at the same time, variations in individual Working Memory Capacity may impact performance and mental workload during demand transitions, and such variations may be incorporated into ACT-R for more accurate modeling. Second, the level of automation within the multitasking environment may be manipulated offering control for domain-general attentional resources. A domain-general theory of selective attention posits that the control of attention in one content domain can be affected by memory load on another content domain (Lin and Yeh, 2014). For example, the memory load on a primarily visuospatial task may affect the control of attention on a primarily auditory or phonological task. Since multitasking environments often demand attention from multiple domains, controlling specific domains may help to further clarify cognitive modeling and hysteresis effects when an individual is presented with specific types of tasks or a combination of specific types of tasks. Future research could involve the use of a control group with subjects randomly designed to either an experimental condition or a control condition with a continuous level of difficulty. Such research could also use training performance criterion to ensure that all subjects achieved the same minimal level of competency with the AF-MATB tasks before moving on to an experimental or control session. Future studies should attempt to improve and validate the ACT-R model to predict and improve human performance and cognition for workload transition effects within a multitasking environment.

## Conclusion

By combining ACT-R modeling and EEG with effective connectivity analysis, this study demonstrated how researchers may identify and analyze the effects of workload transition. These findings on the neural mechanisms of hysteresis effects in multitasking environments may be applied to future neuroergonomics research. Neuroergonomics seeks to expand the theoretical and applied frameworks of human factors and ergonomics. By identifying the effects of workload transitions induced by transient events of high workload or low workload, researchers can begin to develop cognitive training programs for specific cognitive abilities and adaptive aiding. The combination of ACT-R modeling and EEG paves the way toward the efficient use of brain network patterns for cognitive state parameters and may expedite the implementation of user-adaptive systems in ecological settings.

## Ethics Statement

All procedures were approved by a University Institutional Review Board of North Carolina State University (protocol #12512) and participants gave their written consent after a detailed explanation of the experimental procedure which was reviewed and approved by the University’s Institutional Review Board.

## Author Contributions

All authors contributed to the conception and design of the study and to the interpretation of the findings of the study. NK and RH collected the data. NK analyzed the data. NK, RH, and CN drafted the manuscript.

## Conflict of Interest Statement

The authors declare that the research was conducted in the absence of any commercial or financial relationships that could be construed as a potential conflict of interest.
